# A Case of Obstinate and Prolonged Rigidity, with Spasmodic Contraction of the Neck of the Uterus during Parturition, Treated by the Combined Administration of Ergot and Chloroform

**Published:** 1853-08

**Authors:** Napoleon B. Anderson

**Affiliations:** Louisville, Ky.


Art. III.—A *case af obstinate and prolonged rigidity, with spasmodic contraction of the neck of the Uterus during parturition, treated by the combincd administration of Ergot and Chloroform.* By Napoleon B. Anderson, M. D., of Louisville, Ky.

During the past few months, as several cases similar to the one in question have come under my treatment, (only less obstinate both in duration and intensity, but in which the same agents were employed with the like good results,) I am disposed to publish the annexed case from the fact that similar circumstances have never come under my own observation where the adoption of these remedial agents was so applicable, and resulted in so precise an action as their administration was wont to fulfil.

Ergot, I can but suppose, as having no direct influence in the dilatation of the mouth of the uterus, its action being secondary, it is a mechanical pressure exerts through the agency of the fœtus—whilst chloroform has the power of effecting this object—and where there exists a stubborn condition of the neck towards dilatation, with inefficient pains, there does not exist more safe, though potent remedies, by which the difficulties can be overcome, than the timely and judicious use of ergot and chloroform, provided there exists a proportionate size of the pelvis, with proper relaxation of the soft parts; and when the administration of these agents does result unfavorably in their action, the consequences will be found in every case to be the result of unforseen barriers existing in the formation of the pelvis and soft parts, or the idiosyncrasy of the patient forbidding their use.

On Sunday morning, June 19th, I was in attendance on Mrs. Mary Finley, of this city, then in labor with her second child. She is twenty-four years of age, possessing an unusually healthy constitution, being well proportioned in every respect. On my arrival, I was informed that she had been in labor the previous twenty-four hours, the pains having been small but regular. On a vaginal examination it was found impossible to reach the mouth of the womb in almost any position of the patient, and it was only after repeated trials that it was brought down by elevating the hips high in bed. The position of the os uteri was found to be backward, upward, and to the left side, imparting to the touch a sensation similar to that produced when pressure is made on the cartilage of the end of the nose. At each successive pain it was thrown back in the same position, and it was found almost impossible to retain it in the axis of the pelvis. Finding it impossible to control the position of the neck, she was suffered to remain unexamined for several hours, during which interval the neck righted itself, but remained uninfluenced by the pains which were materially augmented. The extract of belladonna was freely used on the neck, which, in some five hours, produced but slight dilatation, and the remedy soon lost its influence, the parts remaining as hard and obstinate as in the commencement of labor. The pains soon became constant and expulsive, but were attended by no beneficial result, as the parts refused to yield to the hardest pains. The patient became flushed, excited, and experienced great pain in the head, for which she was freely bled, but producing no beneficial results so far as dilatation of the mouth of the uterus was concerned. Antimony was then resorted to, and she was brought under its full influence, without the slightest effect. The finger was then made an agent by friction constantly kept up around the neck of the uterus until its influence produced slight dilatations, which soon, however, lost its effect. The lips were flabby, yet hard, and the stoutest pains seemed to have no direct influence whatever in accomplishing the requisite enlargement to enable the birth of the child.

After exhausting a great amount of her energy, the pains grew less frequent, feeble, and finally abandoned her almost entirely. She remained in this condition several hours, when the pains again recurred, though weak and regular; and as the soft parts were readily yielding, she was brought under the influence of ergot in 20 grain doses every twenty minutes, until she had taken one and a half drachms. She was soon under its full influence, but the womb positively refused to yield to the most regular and hardest pains, the parts rather seeming to contract.

Her critical condition prompted a resort to chloroform, the influence of which she was soon under, and whilst the pains seemed in no manner interrupted, dilatation immediately ensued, and after a few pains the child's head was born. The uterus instantly, however, contracted tightly around the neck of the fœtus, without the slightest cessation of the pains, and no manual manipulations, used with regard to the safety of the child or future health of the mother, could have assisted in the birth. The chloroform was again repeated, and resulted in the same happy effect.

Anticipating a sudden contraction upon the placenta after the birth of the child, the hand was immediately introduced into the uterus, whilst the attendants removed the child. Yet, notwithstanding this precaution, the contractions were so severe and spasmodic as to require her to be kept under the influence of chloroform until its removal, which was accomplished with the utmost difficulty. The after-pains were prolonged and severe, and the lochia soon ceased to flow; the former was alleviated by anodynes, and the latter by emollient poultices combined with camphor. After the usual period, she again recovered, and now enjoys excellent health.

The above case, it is reasonable to suppose, had it not been for the ergot and chloroform, would have terminated in the death of the fœtus and probably the mother, or of both, as all the remedial agents generally used were faithfully put in requisition, with the exception of instrumental interference, which would have been criminal to have attempted. It may be questioned as to which of the agents the dilatation of the os uteri is due, or as to whether their combined influences were not the exciting cause. Be this as it may, I feel fully assured that had not ergot been given after the pains became inefficient, the birth would not have been accomplished but by the aid of instruments, the use of which, however, would have been out of the question in this case; and I am confident that but for the use of chloroform, preceded as its administration was by ergot, the consequences might have been serious to both mother and child, from the effects of a rigid undilating uterus. The case is clear, to my mind, as one of the remarkable influences and effects of these two agents, and I feel gratified at having been the means of their use in this case, as I am sure but for their administration, two more beings would have been numbered with those that have preceded us.

Louisville, July, 1853.

